# Use and Misuse of C_q_ in qPCR Data Analysis and Reporting

**DOI:** 10.3390/life11060496

**Published:** 2021-05-29

**Authors:** Adrián Ruiz-Villalba, Jan M. Ruijter, Maurice J. B. van den Hoff

**Affiliations:** 1 Department of Animal Biology, Faculty of Sciences, Instituto Malagueño de Biomedicina (IBIMA), University of Málaga, 29080 Málaga, Spain; adruiz@uma.es; 2 BIONAND, Centro Andaluz de Nanomedicina y Biotecnología, Junta de Andalucía, Universidad de Málaga, 29590 Málaga, Spain; 3 Department of Medical Biology, Amsterdam University Medical Centres, Location Academic Medical Center, Meibergdreef 15, 1105AZ Amsterdam, The Netherlands; j.m.ruijter@amsterdamumc.nl

**Keywords:** qPCR analysis, C_q_, quantification cycle, quantification threshold, PCR efficiency, Poisson variation, LOD, LOQ

## Abstract

In the analysis of quantitative PCR (qPCR) data, the quantification cycle (C_q_) indicates the position of the amplification curve with respect to the cycle axis. Because C_q_ is directly related to the starting concentration of the target, and the difference in C_q_ values is related to the starting concentration ratio, the only results of qPCR analysis reported are often C_q_, ΔC_q_ or ΔΔC_q_ values. However, reporting of C_q_ values ignores the fact that C_q_ values may differ between runs and machines, and, therefore, cannot be compared between laboratories. Moreover, C_q_ values are highly dependent on the PCR efficiency, which differs between assays and may differ between samples. Interpreting reported C_q_ values, assuming a 100% efficient PCR, may lead to assumed gene expression ratios that are 100-fold off. This review describes how differences in quantification threshold setting, PCR efficiency, starting material, PCR artefacts, pipetting errors and sampling variation are at the origin of differences and variability in C_q_ values and discusses the limits to the interpretation of observed C_q_ values. These issues can be avoided by calculating efficiency-corrected starting concentrations per reaction. The reporting of gene expression ratios and fold difference between treatments can then easily be based on these starting concentrations.

## 1. Introduction

**Figure 1:**
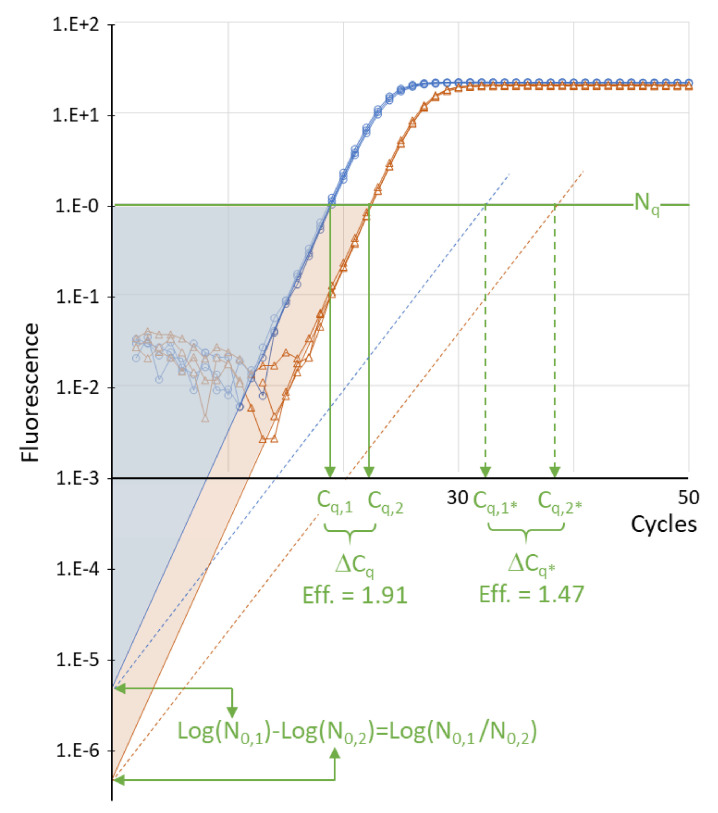
Definition of C_q_ and ΔC_q_. When amplification curves are plotted on a logarithmic fluorescence axis, the exponential phase, with a constant PCR efficiency, can be identified as the cycles where the fluorescence values are on a straight line (solid lines; Eff. = 1.91). The intercept of the downward extrapolated line with the *Y*-axis is the fluorescence associated with the starting concentration (N_0_). Setting a quantification threshold (N_q_, horizontal green line) is used to determine the C_q_ value, defined as the number of amplification cycles needed to reach the threshold (vertical green arrows). The uniform blue and orange triangles, from N_0_ to N_q_ to C_q_, illustrate that for two sets of reactions with about 10-fold different starting concentrations and the same PCR efficiency; the difference in C_q_ values is proportional to the difference in log(N_0_). Therefore, the difference in C_q_ values (ΔC_q_ = C_q,1_ - C_q,2_) is proportional to the log of the starting concentration ratio (log(N_0,2_/N_0,1_). The dotted lines illustrate that the same ratio of N_0_ values and an assay with a lower PCR efficiency (Eff. = 1.47) lead to higher C_q_ values (C_q,1*_ and C_q,2*_) and a larger ΔC_q*_. The graph is based on actual amplification data; the dotted lines represent a hypothetical less efficient assay.

The quantitative polymerase chain rection (qPCR) is based on the real-time monitoring of the fluorescence increase per cycle during the amplification of DNA. This fluorescence is generated by a DNA binding fluorochrome upon binding to double-stranded DNA, a fluorophore released by digestion of a probe during elongation of the primers or by a fluorochrome bound to a probe that fluoresces after binding to the target during DNA synthesis [1]. The quantification cycle or C_q_ value of an amplification reaction is defined as the fractional number of cycles that were needed for the fluorescence to reach a quantification threshold (Figure 1) [2]. 

The more copies of target in the input of the reaction, the fewer cycles of amplification are needed to reach the amount of amplification product associated with the quantification threshold [3]. This simple relation forms the basis of the original qPCR data analysis, which still is the starting point of most current qPCR analysis methods [2,4]. The results of qPCR measurements are most often reported based on the view that the observed C_q_ is solely dependent on the starting concentration of the target. Because of this view, qPCR results can be reported as ΔC_q_ and ΔΔC_q_ values, which supposedly represent the gene expression ratio and fold change between experiments, respectively [5]. This way of reporting of qPCR data is an unintended consequence of applying the simplification of qPCR calculations proposed in the early years of qPCR [6] without addressing the requirements, equality of thresholds and similarity in PCR efficiencies, that allow the use of this simplification. Retrospective evaluation revealed that most papers did not check the validity of the simplification required to report C_q_ values, and do not even enable the readers to do so [2,7].

The guidelines for the Minimal Information for Publications on Quantitative Real-Time PCR Experiments (MIQE) give a checklist of essential and desirable information that should be reported to enable the reviewer to judge the validity of the paper and the reader to repeat the experiment and reproduce the results [2]. With respect to the reporting of qPCR results as only C_q_ values, MIQE already states that “the most popular method is not necessarily the most appropriate” and acknowledges that, when PCR efficiencies differ between assays, the “calculations of relative concentrations will be inaccurate”. Although MIQE is thus showing reticence with respect to reporting qPCR results as only C_q_ values, it also does not clearly advise against this common practice. In a survey that was carried out five years after the publication of the guidelines, the procedures reported in papers were still considered inadequate and therefore likely to generate questionable results [7]. Specifically, “lack of information regarding PCR efficiency” was mentioned as a serious omission because “small differences in this parameter can result in substantial shifts to the quantification cycle (C_q_)”. This overall lack of technical and quality-control details makes it difficult to assess the biological or clinical relevance of the published results [7].

In this review, we will describe and discuss how reported qPCR results are biased by ignoring the dependence of C_q_ on amplification efficiency, how the setting of the quantification threshold leads to differences and variability in the observed C_q_ values and how unavoidable pipetting errors and sampling variation as well as PCR-affecting contaminants hamper the interpretation of reported C_q_ values. Taken together, these issues can all vary between samples, assays, plates and laboratories. As a consequence, a meaningful interpretation of the results of a qPCR experiment is only possible when all of these factors are taken into account. It is of relevance to note that the scope of this paper is limited to the qPCR proper. We will, therefore, not discuss effects of pre-PCR steps, such as sample collection, purification and yield of the RT reaction, although these procedures also affect the observed C_q_ value [8,9,10]. In most sections of this paper, we will assume that the PCR only amplifies the intended specific product, without amplification of artefacts. However, amplification of artefacts may contribute to the observed fluorescence and thus lower the C_q_ value [11]. Therefore, in Section 8, the relation between C_q_ and artefact amplification will be discussed. That section shows that for correct interpretation of C_q_ values, the amplification of artefacts should be checked and, if present, the reactions should be excluded [12]. In the development and validation of a qPCR assay, these confounding factors should already have been carefully considered [2,13]. Finally, the paper will focus on the clinical implications of the factors affecting the observed C_q_ and the quantitative and diagnostic interpretation of observed C_q_ values. 

## 2. C_q_ and the Basics of qPCR

The basic equation for the kinetics of PCR states that the number of target copies (N_c_) after (c) cycles is the starting number of target copies in the reaction (N_0_) times the amplification efficiency (E; defined as fold increase per cycle and ranging from 1 to 2) to the power the number of amplification cycles (c):

(1) $$N_{c}=N_{0}E^{c}$$

The logarithmic form of this equation reads:

(2) $$\log\left(N_{c}\right)=\log\left(N_{c}\right)+c\log\left(E\right)$$

Equation (2) shows that when plotted on a logarithmic fluorescence axis, the exponential phase of the amplification curve is a straight line with slope log(E) (Figure 1). The reader should note that on a linear fluorescence scale, the exponential phase of the reaction is found where the fluorescence starts to rise in the lower limb of the S-shaped curve; its position and length can hardly be evaluated.

When the amplification reaches the quantification threshold (N_q_), Equation (1) reads

(3) $$N_{q}=N_{0}E^{C_{q}}$$

Because C_q_ cycles were needed for the fluorescence of the amplified product to reach the threshold, the logarithmic form of Equation (3) can be rearranged into an equation that shows the dependencies of C_q_ (Equation (4)).

(4) $$C_{q}=\left(\log\left(N_{q}\right)-\;\log\left(N_{0}\right)\right)/\log\left(E\right)$$

The latter equation shows that the C_q_ value of a reaction is not only determined by the target concentration (N_0_), but also by the PCR efficiency (E) [14] as well as the level of the quantification threshold (N_q_).

## 3. Simple Quick Interpretation of C_q_

A simple way to interpret a given C_q_ value in terms of input copy number was previously presented [15]. This method is based on the commonly accepted rule of thumb that, with an input of 10 template copies in the reaction and a PCR efficiency between 1.8 and 2, a C_q_ value of approximately 35 will be observed [16]. In short, with 1 pmol of primers in the reaction, which equals 6.022 × 10^11^ primer molecules, and a PCR efficiency of 1.9, it takes 38 cycles to amplify 10 target molecules into the number of amplicon copies that comes close to the number of primer molecules still present in the reaction. However, competition between primers and single-stranded amplicon during annealing will start about three cycles earlier when the amplicon number reaches 90% of the initial primer concentration [17]. The resulting competition will decrease the PCR efficiency, observed as the transition of the reaction into the plateau phase. Therefore, a reaction that starts with 10 copies of template will show a C_q_ value of approximately 35 cycles when N_q_ is set near the end of the exponential phase [15]. According to this rule of thumb, for 10 copies of template, Equation (3) can be written as $N_{q}=10\times E^{\left(35\right)}.$ When we combine this equation with Equation (3) for an unknown *N* and observed C_q_, cancelling out N_q_ and rearrangement shows that the unknown target quantity can be calculated with:

(5) $$N=10\times E^{\left(35-C_{q}\right)}$$

With Equation (5), one can easily calculate that an observed C_q_ value of 30 and PCR efficiency of 1.8 can be interpreted as the presence of 10 × 1.8^(35−30)^ = 189 copies of target at the start of the PCR. 

## 4. Calculations with C_q_

Equation (4) and Figure 1 show that C_q_ is a function of log(N_0_). Moreover, the difference of two C_q_ values, often written as ΔC_q_, is commonly accepted as the exponent of the simplified equation to calculate the gene expression ratio [6]. These usages of C_q_ and ΔC_q_ raise the question of which mathematical operations can be carried out with C_q_.

### 4.1. The Difference between Two C_q_ Values

The comparison of the two downward-extended amplification curves in Figure 1 results, on a logarithmic scale, in two uniform triangles, showing that the difference in C_q_ values is proportional to the difference in the logarithm of the target quantities. With Equation (4), the difference of two C_q_ values for the same gene can be written as:

$$C_{q,1}-{\;C}_{q,2}=\left(\log\left(N_{0,2}\right)-\;\log\left(N_{0,1}\right)\right)/\log\left(E\right)$$

When ΔC_q_ is defined as C_q,1_-C_q,2_, this equation reads:

$$\Delta C_{q}=\left(\log\left(N_{0,2}\right)-\;\log\left(N_{0,1}\right)\right)/\log\left(E\right)$$

which shows that ΔC_q_ is dependent on the PCR efficiency (Figure 1, solid and dotted lines).

Using the logarithm quotient rule, stating that the differences of two logarithms is the logarithm of the ratio of their arguments, this equation converts into

$$\log\left(N_{0,2}/N_{0,1}\right)=\Delta C_{q}\times \log E$$

Therefore, the expression ratio of two targets can be calculated with:

(6) $$\mathrm{Ratio}\left(N_{0,1}/N_{0,2}\right)=E^{-\Delta C_{q}}$$

Equation (6) shows that, provided that the quantification threshold and PCR efficiency are the same for the two targets, or when two reactions measured the same target, the difference in C_q_ values, ΔC_q_, can be used to calculate the gene expression ratio [6]. 

### 4.2. Averaging C_q_ Values

Averaging C_q_ values involves summation and dividing by the number. Whereas the difference of C_q_ values indicates division of target quantities, the logarithm product rule will effectively state that summation of C_q_ values indicates multiplication of target quantities. Although simple multiplication of target concentrations would be a senseless operation, in the context of calculating the mean C_q_ value, the summation of C_q_ values turns out to be a valid procedure, as shown in the following series of equations:

(7) $$\sum \left(C_{q,i}\right)/N≅\sum \left(\log\left(N_{0,i}\right)\right)/N\;\;\sum \left(C_{q,i}\right)/N≅\log\left(\prod \left(N_{0,i}\right)\right)/N\;\sum \left(C_{q,i}\right)/N≅\log\left(\sqrt[{N}]{\prod \left(N_{0,i}\right)}\right)$$

The argument of the logarithm on the right side of Equation (7), the *n*-th root of the product of n values, is defined as the geometric mean of target quantities. Thus, the arithmetic mean of the C_q_ values represents the logarithm of the geometric mean of the associated target quantities (N_0_). This relation justifies the use of the mean C_q_ of technical or biological replicate measurements in calculations of gene expression ratios or fold difference [14]. In the calculation of such gene expression ratios, the use of the geometric mean is recommended because the geometric mean assures that very different N_0_ values all contribute proportionally to the “mean” value used in the calculation; this is especially important when a set of reference genes with different expression levels is used in gene expression analysis [18]. However, because the biological N_0_ is assumed to be normally distributed, the average gene expression of technical replicates per sample, or biological replicates per experimental condition, can be calculated as the arithmetic mean; such use of the arithmetic mean will simplify the statistical comparison between sample groups [19].

### 4.3. Ratio of C_q_ Values

Some qPCR papers report gene expression ratios as the ratio of the observed C_q_ values [20]. Dividing the C_q_ value of target A by the C_q_ of target B is equivalent to dividing the logarithms of the starting concentrations of these two targets. Note, however, that the ratio of logarithms is not the same as the above discussed logarithm of their ratio. Even worse, the ratio of logarithms, as well as their product, has no mathematical or biological meaning. E.g., in reaction conditions where 10 copies in the reaction result in a C_q_ value of 35, a 10-fold difference in gene expression, 10 and 100 copies (C_q_ values 35.0 and 31.4 cycles, respectively), would give a C_q_ ratio of 0.897 (=31.4/35.0). Whereas, in similar conditions, reactions with 100 and 1000 copies (C_q_ values 31.4 and 27.8 cycles, respectively) would give a similar, but different, C_q_ ratio of 0.886 (=27.8/31.4). However, because there is no mathematical way to convert a C_q_ ratio into a gene expression ratio, this small difference cannot be interpreted as similar expression ratios. Statistical comparison of C_q_ ratios between samples would, therefore, also be meaningless. Calculating the ratio of two C_q_ values, or, similarly, dividing or multiplying C_q_ values with a certain factor, makes no sense, and should therefore never be used or reported in qPCR data analysis. Note that although the averaging of C_q_ values has biological meaning (see Section 4.1), the calculation of C_q_ variation involves multiplication and division, and, therefore, the reporting of C_q_ variation is never appropriate [2].

### 4.4. Between-Plate Correction by Dividing Cq Values

**Figure 2:**
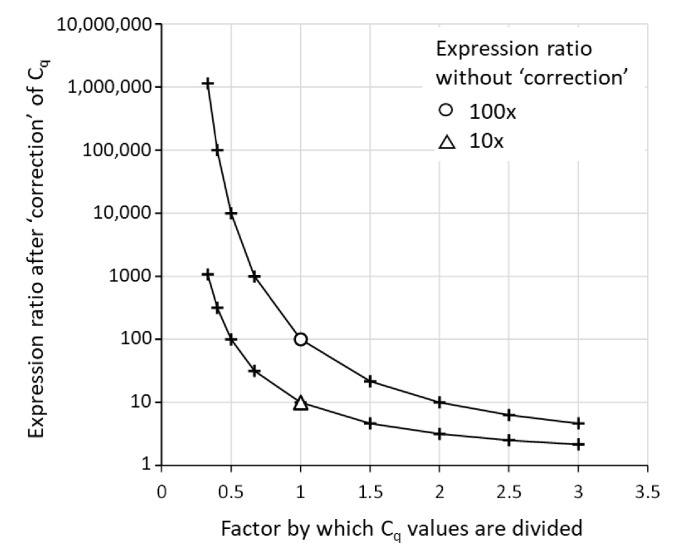
Incorrect use of C_q_ for removal of between-plate variation. Some methods to remove between-plate variation are erroneously based on dividing the C_q_ values on a plate by a factor per plate. The graph is based on hypothetical samples with three true expression levels (N_0_ values = 10, 100 and 1000) measured on nine plates with between-plate differences ranging from 0.33 times down to 3 times up. The measured expression levels were converted into C_q_ values, divided by the factor per plate and re-converted into “corrected” expression levels. Between-plate correction should preserve the expression ratios on each of the plates in the experiment. Therefore, the 100- and 10-fold ratio between the highest and the middle N_0_ (circle) and the highest and the lowest N_0_ (triangle), should not be preserved. The graph shows that dividing C_q_ values will lead to distortion of expression ratios.

A qPCR experiment that requires multiple plates often shows a multiplicative between-plate variation—all measured values on a plate are increased or decreased with a constant factor. C_q_ values have been used to remove this systematic variation between plates in different runs in a qPCR experiment. However, this use of C_q_ values is wrong. To remove between-plate variation, several correct and valid methods have been recommended [19,21]. These between-plate correction procedures utilize replicate measurements of specific calibrators, or any sample with biological or technical replicates, on every plate. To remove between-plate variation, a correction factor for each plate in the experiment is determined from the measured target quantities (N_0_) of these replicates. Dividing all observations on the plate by this correction factor will effectively remove the between-plate variation without affecting the gene expression differences on the plates. However, the reader should note that this approach cannot be applied to the observed C_q_ values. Because of the logarithmic relation between C_q_ value and target quantity (Figure 1), dividing all C_q_ values on a plate by a constant factor per plate will distort the target quantity profile on the plate (Figure 2). To avoid the dissemination of such a wrong correction approach, no references to such misuse of C_q_ values are given. 

## 5. Interpretation of Reported C_q_ Results Is Biased Due to Ignoring the PCR Efficiency

Despite warnings that when PCR efficiencies differ between assays, the ΔC_q_ calculations of relative gene expression concentrations will be inaccurate [2], the reporting of C_q_, ΔC_q_ and ΔΔC_q_ is still commonplace in qPCR papers. Because PCR efficiencies are almost always less than 2, and often different between assays, interpreting the reported C_q_ values as gene expression levels introduces a significant bias in the assumed biological effects.

### 5.1. Bias in Target Quantity 

Quantitative PCR is primarily used to determine the starting concentration, or target quantity, of a DNA or RNA target of interest in biological or clinical samples. Rearrangement of Equation (3) shows that the efficiency-corrected target quantity (N_0_) for each reaction can be calculated with the quantification threshold, the actual PCR efficiency and the C_q_ value observed in the reaction [22,23]:

(8) $$N_{0}=N_{q}/E^{C_{q}}$$

When only C_q_ values are reported in a paper, the reader has to interpret these values by assuming that the PCR was 100% efficient. Because this is never the case, the interpretation will be biased [5]. When an efficiency of 100% is assumed (E = 2), Equation (8) reads $N_{0}=N_{q}/2^{C_{q}}$. Because of this assumption, the biological interpretation of the reported C_q_ is biased. In this paper, this bias is defined as the target quantity calculated with an efficiency of 2 divided by the target quantity calculated with the actual efficiency (Equation (8)). This bias is described with Equation (9).

(9) $$\mathrm{Bias}=\left(N_{q}/2^{C_{q}}\right)/\left(N_{q}/E^{C_{q}}\right)=-1/{\left(\frac{2}{E}\right)}^{C_{q}}$$

**Figure 3:**
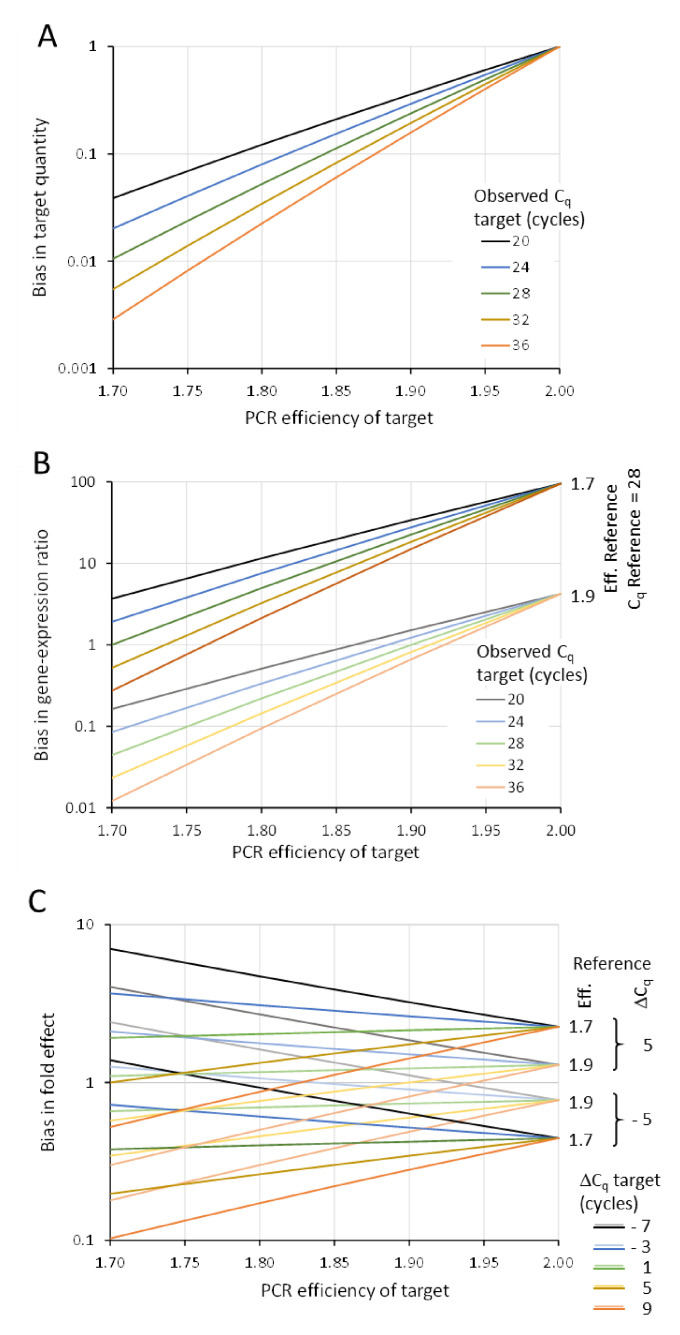
Biased interpretation of reported C_q_, ΔC_q_ and ΔΔC_q_. Reported C_q_, ΔC_q_ and ΔΔC_q_ values require the reader to interpret these results as if all PCR assays are 100% efficient (Eff. = 2). The bias introduced by this interpretation is defined as the assumed result divided by the result that should have been calculated with the actual PCR efficiencies. (**A**) Biased gene expression by interpreting C_q_ as if the PCR efficiency is 100% (Equation (9)). (**B**) Biased gene expression ratio by interpreting ΔC_q_ as if the PCR efficiency of both assays is 100% (Equation (10)). (**C**) Biased fold effect between experimental conditions by interpreting ΔΔC_q_ assuming that the PCR efficiency of both assays is 100% (Equation (14)).

Figure 3A illustrates the bias calculated using Equation (9) for different C_q_ and PCR efficiency values. Because the actual efficiency is always lower than 2, the interpreted target quantity is always an underestimation. The graph shows that at a C_q_ of 28 and an actual efficiency of 1.7, the target quantity calculated with an efficiency of 2 is 100 times lower than it actually is (Figure 3A, asterisk). This example shows that, especially with high C_q_ values, assuming an efficiency of 2 will give the false impression of a very sensitive assay. From this graph, it is immediately evident that a single C_q_ value, often advertised as indication of assay sensitivity, is meaningless. Unbiased interpretation will only occur when the actual PCR efficiency of the target is 2 (Figure 3A, black dot).

### 5.2. Bias in Target/Reference Ratio

In qPCR research, differences in sample size and sample composition are normalized by dividing the target quantity of the gene of interest by that of one, or preferably multiple, reference gene. The latter are genes that are not affected by the experimental or clinical conditions [18]. The normalized gene expression in a sample is then calculated as the ratio of target quantities: $N_{0,\mathrm{tar}}/N_{0,\mathrm{ref}}$. With the substitution of Equation (8), and assuming the same N_q_, this normalized gene expression becomes $E_{\mathrm{ref}}^{C_{q,\mathrm{ref}}}/E_{\mathrm{tar}}^{C_{q,\mathrm{tar}}}$. When the actual PCR efficiencies are ignored and both replaced by an assumed amplification efficiency of 100% (E = 2), this gene expression equation can be simplified to $2^{-\Delta \mathrm{Cq}}$, where ΔC_q_ stands for the difference in C_q_ values (C_q,tar_ − C_q,ref_) [6]. When no PCR efficiencies are reported, the reader can only interpret the reported ΔC_q_ as representing a gene expression ratio of $2^{-\Delta \mathrm{Cq}}$. The bias, defined as the interpreted gene expression ratio divided by the efficiency-corrected gene expression ratio, is given by:

(10) $$B\mathrm{ias}={\left(\frac{2}{E_{\mathrm{ref}}}\right)}^{C_{q,\mathrm{ref}}}/{\left(\frac{2}{E_{\mathrm{tar}}}\right)}^{C_{q,\mathrm{tar}}}$$

Equation (10) shows that this bias depends on the efficiency and C_q_ values of the target and the reference. The graph of this bias for different PCR efficiency values of the target and different C_q_ values of the target was plotted for references with efficiency values of 1.7 and 1.9 and a C_q_ value of 28 (Figure 3B). Although the line at a bias = 1 might suggest that there is a whole range of C_q_ and efficiency values that will give unbiased ratios, this is only the result of coincidental combinations of those values. This is the case when the efficiencies are equal and the reported ΔC_q_ value is zero but other, less obvious, combinations also give a seemingly unbiased interpretation. The only true unbiased gene expression ratio would be found when the PCR efficiencies of target and reference are both 2, which never happens. In all other cases, interpretation of the reported ΔC_q_ value as an expression ratio of $2^{-\Delta \mathrm{Cq}}$ will lead to a biased interpretation of the expression ratio. The direction of this bias depends on the difference in PCR efficiencies of the target and the reference and on the value and direction of the ΔC_q_. The graph shows that, depending on the actual efficiencies of target and reference assays and the C_q_ of the target, the bias can range between 100-fold underestimation and 100-fold overestimation of the gene expression ratio (Figure 3B).

### 5.3. Bias in Fold Effect or Treatment/Control Ratio

To calculate the fold change or fold effect induced by a biological condition or experimental treatment, the gene expression ratio in the treated sample is divided by the gene expression ratio in the control sample:

(11) $$\mathrm{Fold}\;\mathrm{effect}=\left(\frac{N_{0,\mathrm{tar},\mathrm{tr}}}{N_{0,\mathrm{ref},\mathrm{tr}}}\right)/\left(\frac{N_{0,\mathrm{tar},\mathrm{co}}}{N_{0,\mathrm{ref},\mathrm{co}}}\right)$$

Substitution of Equation (8) into Equation (11), cancelling out N_q_ and subsequent rearrangement results in the classic equation for efficiency-corrected relative quantification [14]:

(12) $$\mathrm{Fold}\;\mathrm{effect}=\frac{E_{\mathrm{tar}}{}^{{\Delta \mathrm{Cq}}_{\mathrm{tar}}\left(\mathrm{co}-\mathrm{tr}\right)}}{E_{\mathrm{ref}}{}^{{\Delta \mathrm{Cq}}_{\mathrm{ref}}\left(\mathrm{co}-\mathrm{tr}\right)}}$$

In Equation (12), ΔC_q_ indicates the difference between the mean C_q_ values observed in the control and the treatment groups for the target as well as the reference gene. When the efficiencies are not reported, one must assume an efficiency of 100% for both assays, and Equation (12) can be simplified and rearranged into the equation that is commonly known as the ΔΔC_q_ or comparative C_q_ equation:

(13) $$\mathrm{Fold}\;\mathrm{effect}=2^{-\left({\Delta \mathrm{Cq}}_{\mathrm{tar}}\left(\mathrm{co}-\mathrm{tr}\right)-{\Delta \mathrm{Cq}}_{\mathrm{ref}}\left(\mathrm{co}-\mathrm{tr}\right)\right)}$$

Equation (13) has been further simplified to Fold effect = 2^-ΔΔCq^. The two delta symbols stand for the difference in the difference in C_q_ values between target and reference gene under the control and experimental conditions [6]. Although never intended, further simplification of this equation has led to the common practice and general acceptance of reporting only ΔΔC_q_ to represent the fold effect found by qPCR analysis. The bias introduced by this interpretation of ΔΔC_q_, defined as the fold effect assuming an efficiency of 2 for all conditions and assays (Equation (13)) divided by the fold effect calculated with efficiency-corrected target quantities (Equation (11)), can be mathematically described as:

(14) $$\mathrm{Bias}={\left(\frac{2}{E_{\mathrm{ref}}}\right)}^{\left(C_{q,\mathrm{ref},\mathrm{tr}}-C_{q,\mathrm{ref},\mathrm{co}}\right)}/{\left(\frac{2}{E_{\mathrm{tar}}}\right)}^{\left(C_{q,\mathrm{tar},\mathrm{tr}}-C_{q,\mathrm{tar},\mathrm{co}}\right)}$$

Figure 3C illustrates the pattern of over- and underestimation of the fold effect for different efficiency values and different ΔC_q_ values for the target in the treated and control sample; the bias is calculated for two efficiency values of the reference (1.7 and 1.9, respectively) and two ΔC_q_ values for the reference in the treated and control sample. As with the gene expression ratio (Equation (10), Figure 3B), the “unbiased” combinations of efficiency and C_q_ values on the bias = 1 line are coincidental combinations of efficiency and ΔC_q_ values. Despite the confusing tangle of lines, the graph shows that in general, a positive ΔC_q_ of the reference gives a positive bias; for a negative ΔC_q_ of the reference, in general a negative bias is observed. In both cases, the bias is larger with lower efficiency of the reference. The magnitude of the biases is less than those of the gene expression ratio (Figure 3B) because the biased interpretation of the gene expression in the samples in the experimental condition is partly compensated by a similar bias in the control condition. However, when large treatment effects are present (a large ΔC_q_ for the target gene between the experimental and the control condition), the fold change interpreted from the reported ΔΔC_q_ can be an up to 10-fold exaggeration of the down-regulation or up-regulation of the target gene (Figure 3C). 

Taken together, the graphs in Figure 3 show that ignoring the actual PCR efficiency in the interpretation of reported C_q_, ΔC_q_ and ΔΔC_q_ data **always** leads to C_q_-dependent biases in assumed biological effects. This biased interpretation of C_q_ values depends on the PCR efficiency values and the PCR efficiency difference between targets and references, which vary between assays and protocols. Ignoring these dependencies might be at the core of the discussions on reproducibility of qPCR experiments. The unbiased reporting of qPCR results requires an analysis of qPCR data that makes use of the actual PCR efficiencies of the amplified targets and reference genes. Only such efficiency-corrected analysis will produce reliable, accurate and reproducible output data per qPCR reaction, sample, target, experimental condition and laboratory.

## 6. Reproducibility and Variability of C_q_

The above considerations show that C_q_ values can only be compared when they are determined with the same quantification threshold. However, the current procedures for setting of the quantification threshold lead to different thresholds and, therefore, to different and variable C_q_ values. Moreover, C_q_ values will also vary when the PCR efficiency differs between reactions.

### 6.1. Threshold Setting and C_q_ Value 

**Figure 4:**
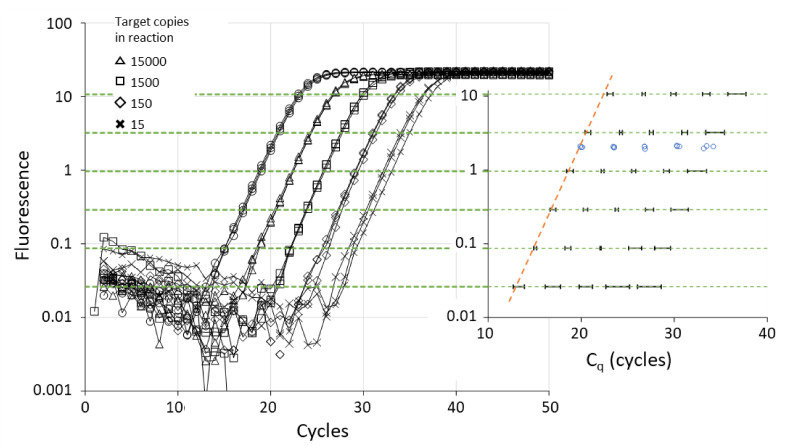
Illustration of relation between threshold level, C_q_ value and C_q_ variability. The graph shows the amplification curves of triplicate measurements of a 5-step, 10-fold dilution series. Quantification thresholds (dotted green lines) were placed at six levels between the ground phase noise and the plateau phase. For each of the amplification curves the C_q_ value was determined. The graph in the inset shows the 95% confidence interval of the observed C_q_ values (mean +/− 2 × SD per dilution). The orange dotted line illustrates that when the threshold is set above the exponential phase, the C_q_ values are higher than expected. The blue dots are the C_q_ values determined from the SDM (see Section 6.2). The graph is based on actual amplification data.

The graph and inset in Figure 4 show that the observed C_q_ values increase linearly when the threshold is set at a higher level. All qPCR machines perform an automatic setting of the quantification threshold, but not all manuals disclose how this is done. In general, the threshold is set at 10 standard deviations (SD) above the mean ground phase fluorescence values. Because these early fluorescence values represent measurement noise and are dependent on the sensitivity setting of the qPCR apparatus, these thresholds can differ between machines and runs and its position in the exponential phase of the reaction should be checked by the user [13]. Because this automatic threshold setting aims to avoid the lowest fluorescence values, there is a risk that the threshold is set above the exponential phase. Because the PCR efficiency in these cycles is declining, this will lead to erroneously high C_q_ values (Figure 4, inset). This risk is exacerbated when a common threshold is set when different assays with different plateau levels are present in the run. To remedy such events, qPCR machines allow the user to manually “adjust” this automatic threshold setting. Although this is done with the correct recommendation that the threshold must be set in the exponential phase, the user may unintentionally introduce additional bias. Especially when the use of the C_q_ values of a dilution series to derive a PCR efficiency value prompts the user to set a threshold such that the C_q_ values in a standard curve give an efficiency value between 1.9 and 2.1.

It is of importance to note that in clinical applications, a sample is often considered positive when the amplification curve reaches the quantification threshold (N_q_) and a C_q_ value is called. In a clinical context, false positives, when the threshold is set too low, or false negatives, when the threshold is set too high, give rise to an incorrect diagnostic decision that might have severe consequences for the patient. Therefore, the analysis procedure should guarantee that the quantification threshold is always set in the exponential phase of the amplification curve [13]. However, with different plateau levels, different PCR efficiencies per assay and variation between samples, the implementation of threshold setting in the software of the qPCR machine does not give this guarantee. For reliable diagnostics, a standardized threshold setting, based on the properties of the amplification curve, is required [5].

Alternatives for the machine software recommend the threshold be set at one cycle below the end of the exponential phase [23], at the mean of the fluorescence of the baseline and the fluorescence at the end of the exponential phase [24] or at the midpoint of the exponential phase [25]. Although these methods guarantee that the quantification threshold is set in the exponential phase, the C_q_ values will still differ between machines, assays and runs. Consequently, C_q_ values cannot be compared between runs, even for the same assay.

### 6.2. Threshold Setting and C_q_ Variability 

Even when the quantification threshold is set in the exponential phase of the reactions, the residual noise in the early cycles of the exponential phase makes it so the variability of the observed C_q_ values is highly dependent on the position of the threshold in this phase (Figure 4, inset). The C_q_ variability increases with lower input in the reactions (Figure 4 from left to right). From the graph, it can easily be seen that with a threshold in the lower part of the exponential phase, there is more C_q_ variability between technical replicates than with a high threshold. Although not obvious in Figure 4, the C_q_ variability may increase when the threshold is set too high. This is most evident when the plateau levels of the different reactions differ; for reactions with a low plateau, the quantification threshold is then set above the exponential phase. In Figure 4, the baseline correction was performed with LinRegPCR [23]; with the baseline trend set by the almost all qPCR machine, the lowest thresholds result in significantly higher C_q_ variation.

To obviate this variation introduced by quantification threshold setting, it has been suggested to define the C_q_ value of a reaction as a fixed point in the reaction kinetics, not at a fixed amount of product. This fixed point in the kinetics is found in the second derivative maximum, SDM for short, defined as the cycle at which the second derivative, showing the acceleration of the fluorescence increase, reaches its maximum, which is always near the end of the exponential phase [22,26]. This SDM value is derived from the fit of a sigmoidal function to the amplification data and reported as C_q_. However, sigmoidal functions do not represent PCR kinetics and were shown not to fit perfectly to the exponential phase of the amplification curves [27]. Moreover, even the smallest increase in fluorescence would allow the algorithm to fit a sigmoidal function and report a C_q_ value even when the amplification curve does not reach the quantification threshold as would be set by the machine or the user. The SDM-based C_q_ is not further discussed in this paper.

### 6.3. C_q_ Variability Because of Differences in PCR Efficiency

**Figure 5:**
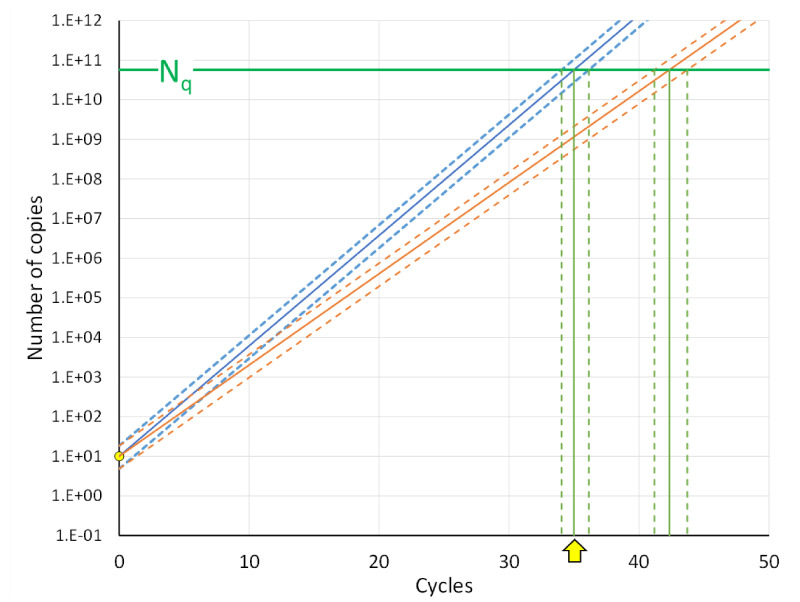
The graph illustrates the kinetic relation between C_q_ value and PCR efficiency for efficiency values between 1.9 (solid blue line) and 1.7 (solid orange line) with an input of 10 copies of target in the reaction (yellow dot). The difference in efficiency values results in a range of C_q_ values between 35 and 42 cycles (vertical, solid light green lines). Because of the Poisson sampling variation, an average input of 10 copies per reaction will actually range between 5 and 18 copies. With the same PCR efficiencies (dotted blue and orange lines) the range of C_q_ values is extended with another 2.5 cycles (dotted green lines). The quantification threshold (N_q_) is set at the level where the 10 copies of the input, with a PCR efficiency of 1.9, result in a C_q_ value of 35 (yellow arrow) [15]. The graph is based on the kinetic equation of PCR (Equation (1)).

When two targets have the same starting concentration but are amplified with different PCR efficiencies, the target with the lowest efficiency will have the highest C_q_ value (Figure 5). Several factors determine the actual PCR efficiency of the assay or the reaction. Apart from the above-discussed technical differences between machines and runs, qPCR experiments also differ with respect to the polymerase enzyme [28,29], intended and unintended reaction mix additives [30], monovalent ion concentrations [31] and primer sequences and concentrations [13]. These factors all affect the PCR kinetics, although these effects are not always consistent and opposite effects were reported for different tissues [32]. Therefore, the C_q_ values from PCR reactions ran under different conditions or with different reagent mixes cannot be compared directly, even when all PCR details are reported [2,7]. This is especially true for additives to the reaction mixture that contribute to destabilization of hairpins in the target DNA and thus affect primer annealing. Moreover, small sequence differences in the primers, or in and around the primer binding sites, affect primer annealing and PCR efficiency [33,34,35,36,37,38]. Though outside the scope of this review, the pre-qPCR steps (sample collection and storage, RNA extraction and reverse transcription) [39] are also sources of variation that have an effect on the C_q_ and PCR efficiency and require optimization. This is particularly evident for point-of-care diagnostics, in which sample purification is beyond the scope, time frame and cost-effectiveness of the analysis. Especially, the omission of sample purification often results in the presence of different contaminating substances which affect the PCR efficiency [40,41]. Therefore, in point-of-care diagnostics, it may be required to use the PCR efficiency of the individual sample, rather than the efficiency of the assay, for meaningful calculation of the observed target quantity [5].

All issues discussed in this section can be resolved by calculation of the efficiency-corrected target quantity (N_0_; Equation (8)) as the primary qPCR result for each reaction in the run. As can be seen in Equation (8), this calculation of the target quantity accounts for the actual threshold setting and PCR efficiency. The latter can be either the PCR efficiency of the assay, or, when individual samples show significantly different PCR efficiencies, the efficiency of the individual sample [5]. After calculating the N_0_ per reaction, the user can, by applying high school mathematics, average the technical replicates and calculate the gene expression ratios per sample ($N_{0,tar}/N_{0,ref}$) and continue the analysis with the calculation of fold differences between conditions (Equation (8)). Because the results of each of those calculations can be visualized, it will be easy to identify deviating reactions, samples and conditions. 

## 7. Effects of Input Variation on Observed C_q_

Apart from the threshold setting and PCR efficiency, C_q_ is mainly determined by the concentration, or number of copies of the target, at the start of the PCR. This copy number in the reaction is determined by pipetting the sample into the reaction well. The variation sources that are thus affecting the actual number of copies in the reaction are the random pipetting error as well as on the statistical sampling variation governed by the Poisson distribution.

### 7.1. Pipetting Error

To avoid systematic pipetting errors, which would affect not only the observed C_q_ values but also the PCR efficiency derived from a dilution series, pipettes used for qPCR analysis should be regularly calibrated [5]. However, random pipetting errors are unavoidable and will affect the accuracy of the observed C_q_ values when interpreted as gene expression in biological samples. When a sample contains an average of N_0_ copies of the target per reaction volume, a fractional pipetting error (P), randomly up or down, will result in an actual starting concentration in the reaction (N) between the lower and upper pipetted input.

(15) $$\left(1-P\right)N_{0}<N<\left(1+P\right)N_{0}$$

**Figure 6:**
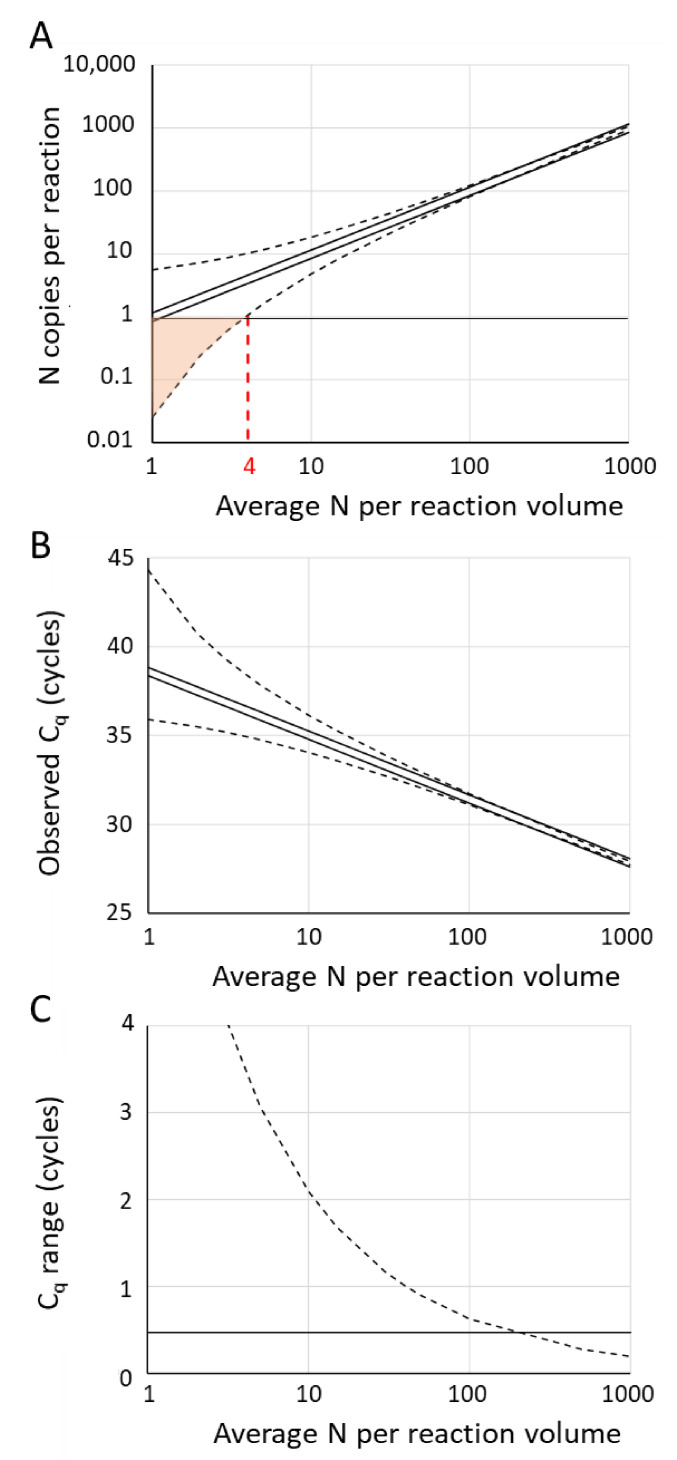
Effect of pipetting error (solid lines, Equation (15)) and Poisson sampling variation (dotted lines, Equation (16)) on the number of copies in the reaction and the observed C_q_ values. (**A**) Actual number of target copies in the reaction. Below an average of four copies (vertical dotted red line), an increasing fraction of the reactions be false negative reactions (pink area). (**B**) Range of observed C_q_ values calculated for an efficiency value of 1.9 and a C_q_ of 35 for an average input of 10 copies. Note that the lower C_q_ values come from reactions with the high number of target copies (panel A). (**C**) Range of C_q_ values for different average number of target copies in the reaction (Equation (17)). The graphs are plotted for a PCR efficiency of 1.9; the effect of the PCR efficiency on the observed C_q_ values is illustrated in Figure 5 and Figure 8.

A graph of Equation (15) for a pipetting error of 15% (P = 0.15) and different average number of copies in the reaction volume is drawn in Figure 6A (solid lines). The parallel lines on a logarithmic scale show that, when only the pipetting error is taken into account, the range of actual input in the reaction is relatively the same for each input and fixed pipetting error (Figure 6A). Because of the logarithmic relation of N_0_ and C_q_, the resulting range of C_q_ values will be the same for every input (Figure 6B).

### 7.2. Poisson Sampling Variation 

When the reaction volume is pipetted from a bulk sample or stock solution into the individual reaction wells, the number of copies present in the different reaction wells will always show a random variation. Because the number of copies in the wells is discrete, independent and random, this number will follow a Poisson distribution. Therefore, pipetting from a stock solution can be described as a sampling process governed by the Poisson distribution and will unavoidably result in a variable actual number of copies in the reaction. The range of the actual number of copies in the reaction can be calculated using the relationship between the Poisson and Chi^2^ distributions [42]. The 95% confidence interval of the actual number of targets (N) in the reaction, therefore, is given by:

(16) $$\frac{1}{2}\chi_{\left(0.025;2N\right)}^{2}\leq N\leq \frac{1}{2}\chi_{\left(0.975;2N+2\right)}^{2}$$

A graph of Equation (16) for different average number of copies in the reaction volume is drawn in Figure 6A (dotted lines). The converging lines on a logarithmic scale in the graph of Equation (16) show that the Poisson variation relatively decreases when the input in the reaction increases (Figure 6A). This leads to a similarly decreasing range of observed C_q_ values with higher input (Figure 6B). When the target number is below four copies per reaction volume, an increasing fraction of the reactions will receive less than one copy of the target and will, therefore, not show amplification; for these reactions, no C_q_ value can be determined (Figure 6A, pink area). 

### 7.3. Combined Effect of Pipetting Error and Poisson Sampling Variation

After substitution of both the upper and lower actual N_0_ of either Equation (15) or Equation (16) into Equation (3), the range of C_q_ values observed between a low and a high input (N_up_ and N_low_, respectively) can be rearranged into the following equation:

(17) $$C_{q,\;\mathrm{range}}=\frac{\log\left(N_{\mathrm{up}}\right)-\log\left(N_{\mathrm{low}}\right)}{\log(E)}$$

For both error sources, this observed C_q_ range is only dependent on the actual range of input quantities and on the PCR efficiency, not on the quantification threshold. As shown in Figure 6B, the pipetting error will lead to a C_q_ range that is the same for every input; e.g., 15% pipetting error and PCR efficiency of 1.9 gives a C_q_ range of 0.5 cycles for all inputs [15] (Figure 6C). However, with decreasing copy number, the Poisson variation dominates the observed C_q_ range; already below 100 target copies per reaction, the C_q_ variation due to Poisson sampling variation becomes larger than the pipetting error (Figure 6C).

In qPCR, technical replicates with C_q_ values that differ more than 0.5 cycles are considered to be too far apart and should be discarded from the analysis [13]. For high copy number samples, i.e., low C_q_ reactions, this 0.5 cycle rejection rule should be applied because such a C_q_ difference results from a 15% pipetting error which should not be ignored (Figure 6C). However, for low copy-number samples, the actual input into the reaction will be dominated by the Poisson sampling variation. Even highly skilled operators will then observe an unavoidable range of more than 0.5 cycles in replicate C_q_ values and would needlessly exclude these reactions from the analysis. For samples with C_q_ values up to 35, depending on the PCR efficiency, a variation between technical replicates up to two cycles should be considered acceptable (Figure 6C) [15]. Precise quantification of these samples, and of samples with even lower copy numbers, will always require more technical replicates. The other option would be to avoid high C_q_ values; increasing the target copy number in the reaction by adding more cDNA input into the reaction may not always be feasible. For instance, to bring an observed C_q_ from 40 down to 35 cycles, one would require about 100 times more cDNA in the reaction.

## 8. C_q_ and Artefact Amplification 

It should be clear by now that direct comparison of C_q_ values between assays, runs and laboratories is in essence not possible or at least should be treated with extreme care. When the PCR efficiency is not taken into account, especially high C_q_ values cannot be interpreted accurately. However, this dependence of C_q_ on PCR efficiency will be ignored when a cut-off on C_q_ is set to distinguish between “positive” and “negative” samples. For qualitative decisions, reaching the quantification threshold, as indication that the reaction shows a required amount of amplification of the target, would be a safer criterion for positivity. However, this criterion is still dependent on the PCR efficiency and on the occurrence of amplification artefacts. 

**Figure 7:**
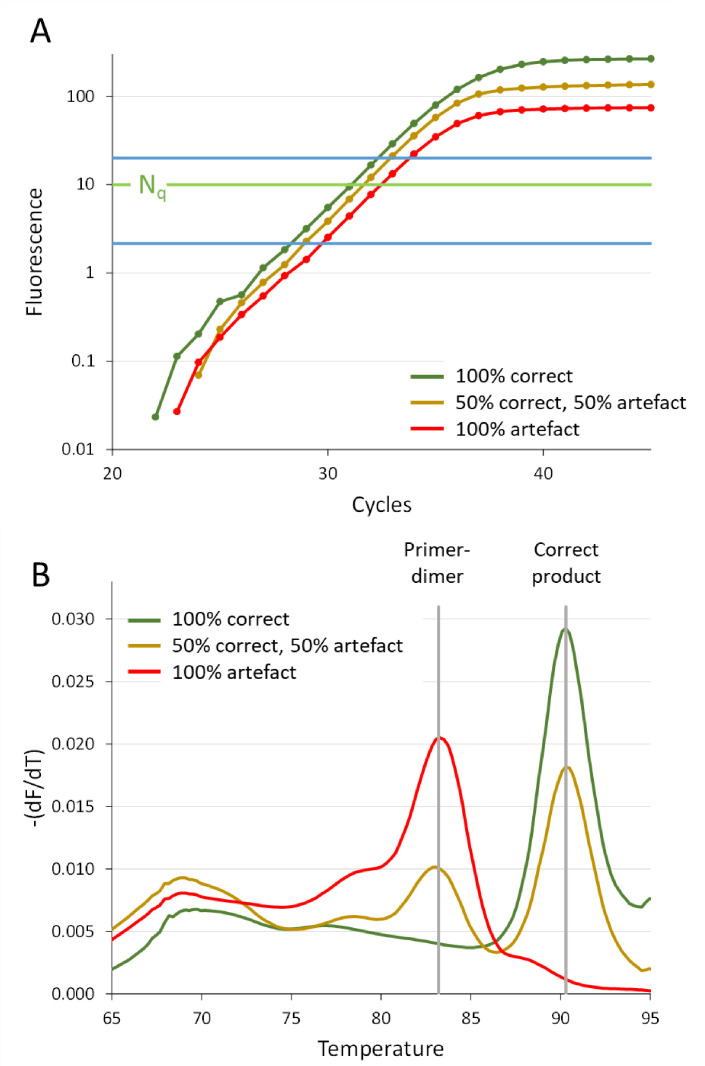
Amplification of correct target and artefact. Different mixtures of intended correct target and primer-dimer artefact were prepared from isolated products and amplified with PCR using the same primer pair for all reactions [11]. (**A**) Amplification curves showing that the PCR efficiency, determined from the selection of data points in the window of linearity (blue lines [23]), is the same for the correct target, the mixture and the artefact [12]. The difference in plateau level reflects the different lengths of the PCR products. (**B**) Graph of the negative first derivative of the melting curves (−dF/dT) reflects the composition of the mixtures that were amplified. The vertical grey lines indicate the melting temperatures of the correct product (90.3 °C) and the primer-dimer artefact (83.2 °C). The presence of the different products in the three reactions illustrates that the observed C_q_ values (panel A, N_q_) are correct (green amplification curve), too low (brown curve) or artificial (red curve). The graphs are based on actual amplification data [11].

Artefacts contribute to the observed fluorescence in PCRs monitored with DNA-binding dye assays. Therefore, amplification of only artefacts may allow the amplification curve to reach above the quantification threshold, resulting in a seemingly positive reaction (Figure 7A). Moreover, when both artefacts and correct products are amplified, the observed C_q_ value is lower than it would have been when only the correct target was amplified (Figure 7), hampering the valid quantification of such a sample. Using a dataset with 93 validated assays, we have shown that amplification of nonspecific products occurs frequently, and that amplification of these products cannot simply be identified from a deviating PCR efficiency or a high C_q_ value, indicated by the slopes and the positions of the amplification curves, respectively [12]. The PCR efficiency observed for reactions that amplify correct products, artefacts or both, are indistinguishable (Figure 7A) [11]. Although the C_q_ value distributions overlapped for all assays, only 5% of the reactions that amplified only correct products had a C_q_ above 34, whereas only 5% of the reactions with a C_q_ under 27 amplified an artefact. Therefore, to stay on the safe side in qPCR analysis, all reactions with a C_q_ above 27 should be carefully checked for the generation of artefacts [12]. 

For valid qualitative and quantitative interpretation of qPCR results, the researcher should make sure that the observed amplification represents the amplification of only the correct specific product. In case of a DNA-binding dye assay, melting curve analysis is a quick and easy way to identify the presence of another than the intended target in the amplification reaction (Figure 7B) [43]. When a saturating dye is used, the contribution of the artefact to the observed fluorescence can be determined from the melting curves and used for correction of the observed C_q_ and N_0_ values [11].

## 9. Diagnostic Interpretation of C_q_ Values 

**Figure 8:**
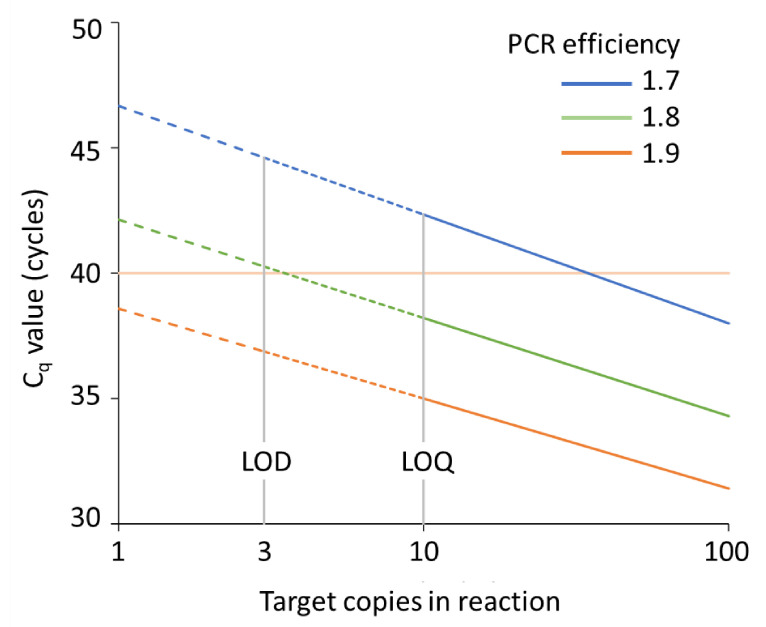
Illustration of the C_q_ values observed for different inputs and efficiency values. The graph shows the observed C_q_ values for different numbers of target copies in the reaction for PCR efficiency values of 1.7, 1.8 and 1.9. The Poisson variation (see Figure 6) means that for inputs below 10 copies the PCR cannot be used for quantitative analysis (dotted lines, LOQ), whereas below three copies even the qualitative (yes/no) conclusion becomes unreliable (dashed lines, LOD; see Section 9.1). However, the graph illustrates that the relation between C_q_ values and LOQ and LOD depends on the PCR efficiency. The MIQE guidelines [2] indicate that the use of an arbitrary cut-off for accepting C_q_ values as valid at, e.g., 40 cycles (pink line), may be either too low (eliminating valid results when the PCR efficiency is low) or too high (increasing unreliable positive results). The graph is based on the kinetic equation of PCR (Equation (1)).

At the start of this paper, we presented a quick quantitative interpretation of C_q_ [15]. The previous sections show that the quantitative accuracy of this interpretation is limited by uncertainty about the PCR efficiency and other sources of variability in the observed C_q_ values. The higher the C_q_ value, the more uncertain the calculated target quantity will become. For very high C_q_ values, the user may even have to be content with a the qualitative “the target is present” answer. However, the opposite answer “the target is not present” becomes questionable when very low target numbers are present in the sample (Figure 8). 

### 9.1. Limits on the Interpretation of C_q_ Values

The limit of detection (LOD) of qPCR is defined as the average number of copies of the target in the reaction that will show amplification in at least 95% of the reactions. Assuming that the PCR will amplify a single copy when it is present, the Poisson sampling variation will limit the LOD to three copies in the reaction with a false negative rate of 3% [2,44]. A similar LOD was found by an experimental approach [45] 

The limit of quantification (LOQ) of qPCR is defined as the number of copies in the sample that can be quantified with a required precision [45]. Although there is no fixed definition of required precision, in general this limit is reached when the variation between technical replicates exceeds the variation between biological replicates. In that situation, replicates would increase biological variation. The technical variation in qPCR is determined by the basic property of the Poisson distribution, which states that the mean copy number in the reactions, observed after replicate sampling from the same stock, will be equal to the variance of these observations. Therefore, with an average of N = 10 copies per reaction, the sampling will result in measurements with a technical standard deviation (SD) of 3.3 and a coefficient of variation (CV = SD/mean) of 33%. In an experimental approach to determine LOQ, a threshold of CV < 35% was found to be reached with 16 molecules [45]. With less input, and thus higher CV, the measurement variation would have an unacceptably large contribution to the total variation in the experiment [46]. Because 10 copies in the reaction result in a C_q_ of about 35 cycles, the Poisson sampling variation will severely hamper the accurate quantification when C_q_ values above 35 cycles are observed. For such low target number samples, technical replicates will always be needed to cancel out the Poisson variation and reach a correct quantitative biological or clinical result. 

**Figure 9:**
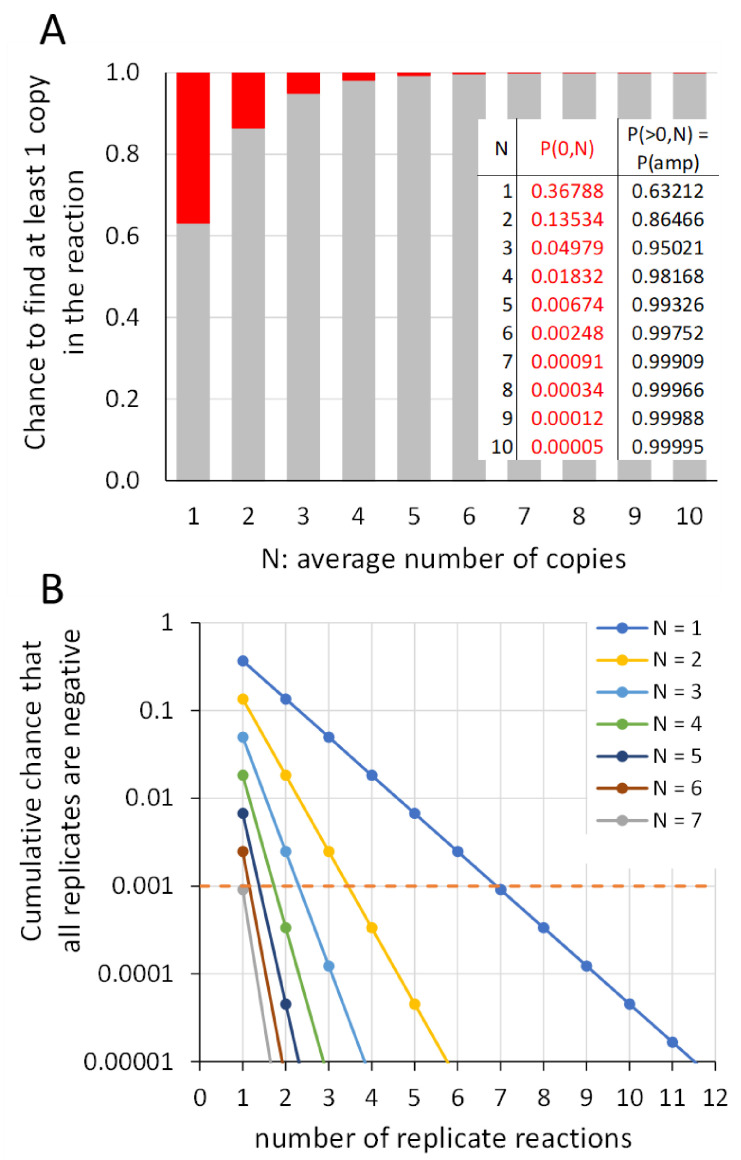
Number of technical replicates needed to declare that a sample is negative. (**A**) Bar graph of the chance, governed by the Poisson distribution, that a PCR reaction contains a target copy and thus shows amplification (grey; P(amp)) or not (red; P(0,N)) for the average number of targets per reaction volume ranging from one to ten. When no amplification is observed, the reaction is a false negative (P(FN)). (**B**) Cumulative P(FN) for a number of technical replicates for different inputs in the reaction (colored lines). The graph shows that in the worst-case situation, i.e., an average input of only one target copy per reaction (blue line), seven negative replicate reactions are needed for the researcher to be 99.9% sure (dashed pink line) that the sample is indeed negative. Note that only one positive replicate is needed to decide that the sample is positive. For samples with on average seven, or more, copies per reaction volume, the chance that a single reaction is positive is already 99.9% or higher.

For the sake of argument, we refer to a published multi-center study, in which two bulk samples with different human herpesvirus 6 load (6000 and 200 copies/mL) were distributed and analyzed by different laboratories with their in-house diagnostic qPCR assays [47]. As should be expected, all laboratories could detect the virus in the bulk sample with a viral load of 6000 copies/mL, whereas only 80% of the laboratories could report a correct qualitative result for the 200 copies/mL bulk sample. Due to the inherent specifications of a qPCR machine, the volume of the assayed sample in the reaction is limited and ranges between 5 and 20 mL in size. In case of the 6000 copies/mL bulk sample, the reactions would then contain 30–120 copies of the target, which both can easily be quantified without significant Poisson variation (see Section 7.2). However, in case of the 200 copies/mL bulk sample, the reactions contain on average only one to four copies of the target. Because of the ever-present Poisson variation, 36% to 2% of the reactions will not receive the target (see Figure 9A, inset table) and will, as a consequence, become false negatives. In case of the 200 copies/mL bulk sample, the volume of the sample pipetted into the reaction should be at least 50 mL (containing on average 10 copies of target) to detect the virus with more than 99.9% confidence in an assay. At this point, it is of relevance to note that the addition of such a large sample volume in a qPCR assay is not possible and that, even in case of just a qualitative outcome, multiple technical replicates would be required (see Section 9.2).

### 9.2. Number of Replicates Needed to Diagnose a Sample as Negative

Because of the unavoidable Poisson sampling variation, the pipetting of samples with low target number will lead to wells without target copies and thus reactions without amplification. When there are, on average, 10 or more target copies per reaction volume present in the analyzed sample, the chance that every reaction shows amplification is very close to 100%; the chance that no copies are present in the reaction is 1 in 100,000 or less (Figure 9A, inset table). For samples that contain less than 10 copies of target per reaction volume, there is an increasing chance (P(FN)) that no copy is present in the reaction and that the reaction is in fact a false negative (Figure 9A). 

How many replicate reactions do we then need to run to be 99.9% confident that a sample is truly negative? Because replicate reactions are independent, the chance that a number (n) of replicate reactions are all false negative is P(FN)^n^. For a sample with on average one copy per reaction volume, two replicate reactions that are both negative would give us 0.368^2^ or only 13.5% confidence that the sample is negative (Figure 9B). Therefore, to be 99.9% confident, seven replicates that show no amplification are required. For samples with more than one copy per reaction volume, the number of required replicates would be lower (Figure 9B). However, because the actual number of targets in the unknown sample is not known, at least seven negative reactions are required. Of course, when one of the technical replicates shows a positive reaction, the sample must be considered positive—assuming, of course, that the researcher can be sure that the positive reaction is not due to the amplification of an artefact which, especially with a low number of target copies in the reaction, occurs frequently [12]. 

## 10. Conclusions 

Taken together, this paper shows comprehensively that differences in usage of materials, qPCR machines, laboratory protocols, analysis procedures and factors affecting the PCR kinetics all lead to differences in the observed C_q_ values within and between experiments. Therefore, C_q_ values cannot be considered to unambiguously reflect the same target concentration for assays, machines, runs and laboratories. Clinical decisions should not be based on C_q_ values alone. At least, the decision which C_q_ cutoff value discriminates between positive and negative reactions should be based on the PCR efficiency of the assay and should be validated per type of material and laboratory. Similarly, the clinical decision that a sample is positive because the amplification curve reaches the quantification threshold cannot be made without considering all factors that affect the PCR kinetics; the data analysis procedure should guarantee that the threshold is always set in the exponential phase of the reactions. 

With respect to quantitative analysis, the reporting qPCR results as C_q_, ΔC_q_ or ΔΔC_q_ values is at best circumspect and at worst pointless. The reporting of C_q_ values must be discouraged—a C_q_ value by itself is meaningless; ΔC_q_ and ΔΔC_q_ are confusing and their interpretation leads to biased notions about the gene expression ratios and between-group effects found in the published experiment. The publication and interpretation of C_q_ values may, therefore, be the cause of the problematic reproducibility of biological effects reached with qPCR assays. As compellingly discussed in this review, the issues with the reported C_q_, ΔC_q_ or ΔΔC_q_ values can easily be avoided by reporting results based on efficiency-corrected target quantities per reaction. This simple approach avoids these biases and provides, moreover, insight into the variation sources at every level of the analysis [5]. 

## Data Availability

Not applicable.
